# The multi-domain exercise intervention for memory and brain function in late middle-aged and older adults at risk for Alzheimer's disease: A protocol for Western–Eastern Brain Fitness Integration Training trial

**DOI:** 10.3389/fnagi.2022.929789

**Published:** 2022-08-18

**Authors:** Yu-Kai Chang, Kirk I. Erickson, Sarah L. Aghjayan, Feng-Tzu Chen, Ruei-Hong Li, Jia-Ru Shih, Shao-Hsi Chang, Chih-Mao Huang, Chien-Heng Chu

**Affiliations:** ^1^Department of Physical Education and Sport Sciences, National Taiwan Normal University, Taipei, Taiwan; ^2^Institute for Research Excellence in Learning Science, National Taiwan Normal University, Taipei, Taiwan; ^3^Department of Psychology, University of Pittsburgh, Pittsburgh, PA, United States; ^4^AdventHealth Research Institute, Neuroscience Institute, Orlando, FL, United States; ^5^Department of Sport Medicine, China Medical University, Taichung, Taiwan; ^6^Department of Biological Science and Technology, National Yang Ming Chiao Tung University, Hsinchu, Taiwan

**Keywords:** *ApoE* gene, BDNF, fitness, meditation, memory, social interaction, brain function

## Abstract

**Background:**

Aging is associated with cognitive decline, increased risk for dementia, and deterioration of brain function. Modifiable lifestyle factors (e.g., exercise, meditation, and social interaction) have been proposed to benefit memory and brain function. However, previous studies have focused on a single exercise modality or a single lifestyle factor. Consequently, the effect of a more comprehensive exercise program that combines multiple exercise modalities and lifestyle factors, as well as examines potential mediators and moderators, on cognitive function and brain health in late middle-aged and older adults remains understudied. This study's primary aim is to examine the effect of a multi-domain exercise intervention on memory and brain function in cognitively healthy late middle-aged and older adults. In addition, we will examine whether apolipoprotein E (*ApoE*) genotypes, physical fitness (i.e., cardiovascular fitness, body composition, muscular fitness, flexibility, balance, and power), and brain-derived neurotrophic factor (BDNF) moderate and mediate the exercise intervention effects on memory and brain function.

**Methods:**

The Western-Eastern Brain Fitness Integration Training (WE-BFit) is a single-blinded, double-arm, 6-month randomized controlled trial. One hundred cognitively healthy adults, aged 45–70 years, with different risks for Alzheimer's disease (i.e., *ApoE* genotype) will be recruited and randomized into either a multi-domain exercise group or an online educational course control group. The exercise intervention consists of one 90-min on-site and several online sessions up to 60 min per week for 6 months. Working memory, episodic memory, physical fitness, and BDNF will be assessed before and after the 6-month intervention. The effects of the WE-BFit on memory and brain function will be described and analyzed. We will further examine how *ApoE* genotype and changes in physical fitness and BDNF affect the effects of the intervention.

**Discussion:**

WE-BFit is designed to improve memory and brain function using a multi-domain exercise intervention. The results will provide insight into the implementation of an exercise intervention with multiple domains to preserve memory and brain function in adults with genetic risk levels for Alzheimer's disease.

**Clinical trial registration:**

ClinicalTrials.gov, identifier: NCT05068271.

## Background

The rapid growth of the aged population has dramatically shifted the global demographic structure (United Nations., 2019). Advancing age has been associated with cognitive decline (Salthouse, 2019), progressive brain deterioration (Li et al., 2020), and increased incidence of various types of dementia, particularly Alzheimer's disease (AD) (Livingston et al., 2020). Considering the significant impact of age-related cognitive decline and AD on individual and societal levels (Roberts et al., 2015; Alzheimer's Association., 2021), as well as the limited curative effects of approved drugs on the progression of AD (Mehta et al., 2017), identifying potentially modifiable lifestyle factors for cost-effective, non-pharmaceutical preventative strategies has been a priority to lessen the adverse impact of aging on cognitive function and brain function.

### Exercise, memory, and brain function

Exercise, a planned, structured, and repetitive form of physical activity that aims to improve or maintain one or more components of physical fitness (e.g., cardiovascular and muscular fitness, balance, coordination, and power) (Caspersen et al., 1985), might be a promising non-pharmaceutical preventative approach for decelerating the trajectory of age-related cognitive decline (Chang et al., 2017b; Northey et al., 2018; Chen et al., 2020a) and the deterioration of brain function (Chen et al., 2020b), as well as for preventing or delaying the onset of AD (Valenzuela et al., 2020). Empirical research has revealed that exercise could beneficially alter the metabolomic profiles related to memory (Gaitán et al., 2021), and greater physical fitness levels associated with exercise have been linked to superior cognitive function (Netz, 2019) and to activation of the prefrontal cortex (Chen et al., 2019). Notably, epidemiological research has indicated that around 2% of dementia cases might be prevented by modifying the lifestyle of physical inactivity (Livingston et al., 2020), reflecting the beneficial role of non-pharmaceutical effects of exercise or higher physical fitness levels.

Exercise has been linked to improvements in working memory (Rathore and Lom, 2017; Chen et al., 2020a), which has been classically characterized as the capacity to dynamically store, update, and manipulate incoming information over short periods of time (Baddeley, 2012), and involves activation of cortical networks including the inferior frontal gyrus, anterior cingulate gyrus, hippocampus, and thalamus (Yin et al., 2013; Gutierrez-Garralda et al., 2014; Toepper et al., 2014). Additionally, deficits in working memory in particular accompany aging and AD (Kirova et al., 2015; Salthouse, 2019), and impaired working memory has been associated with increased psychological disorders (Chai et al., 2018). Fortunately, systematic reviews and meta-analyses have revealed that exercise training, such as aerobic exercise and resistance training, significantly improves working memory performance in cognitively healthy older adults and in older adults with mild cognitive impairment (MCI) (Northey et al., 2018; Chen et al., 2020a). Neuroimaging evidence has further described the beneficial effects of chronic exercise on brain function related to working memory in late middle-aged and older adults, reflected by greater activation in the prefrontal lobe, anterior circulate, and hippocampus (Chen et al., 2019), as well as attenuated aging effects on hippocampal volume (Erickson et al., 2011).

There are fewer studies that have examined the effects of exercise on episodic memory. Episodic memory is long-term, retrospective memory bound to temporal and spatial contexts (Tulving, 2002; Yonelinas et al., 2019) and has been closely linked to the hippocampus and surrounding medial temporal lobe structures (Yonelinas et al., 2019). Using direct (Hayes et al., 2016) or estimated (Boots et al., 2015; Freudenberger et al., 2016) measures of cardiovascular fitness, higher cardiovascular fitness levels have been linked to superior episodic memory in cognitively healthy older adults (Hayes et al., 2016) and late middle-aged adults at risk for AD (Boots et al., 2015). Similar findings have been reported in neuroimaging research. For instance, using the episodic associative learning task, a task that assesses hippocampal-dependent relational binding, Cole et al. (2020) indicated that healthy older adults with higher levels of cardiovascular fitness had larger hippocampal volumes and an enhanced rate of relational memory acquisition. Of note, the protective effects of cardiovascular fitness on episodic memory and brain health might be moderated by sex for individuals at risk for AD, such that positive correlations between cardiovascular fitness and hippocampus volume were observed in older adult women but not men, whereas positive correlations were observed between cardiovascular fitness and episodic memory in older adult men but not women (Dougherty et al., 2017).

### Exercise intervention with multiple exercise modalities, memory, and brain function

Current evidence has highlighted that exercise training or increased physical fitness might function as a non-pharmaceutical strategy against the deleterious effects of aging and AD on memory capacity and brain function; however, it should be noted that the majority of studies have focused on a single exercise modality (e.g., aerobic or resistance training) (Chen et al., 2020a,b). Systematic and meta-analytic reviews have revealed that exercise programs combining multiple exercise modalities (e.g., resistance training combined with aerobic exercise) might potentially evoke even greater benefits on cognitive functions (Kramer and Colcombe, 2018; Tomporowski and Pesce, 2019; Chen et al., 2020a). Notably, health-related physical fitness (e.g., cardiovascular fitness and muscular fitness) and skill-related physical fitness (e.g., balance, coordination, and power) (American College of Sports Medicine., 2018) might impact cognitive functions (Netz, 2019) and activation patterns of the frontal gyrus and premotor cortex regions (Voelcker-Rehage et al., 2010) differentially, reflecting a potential mediating role of type of fitness on cognitive function and brain regions. These results also suggest that the effects of the exercise interventions might vary according to the exercise modalities included in the program. For instance, although an exercise intervention combining aerobic and resistance exercise showed no significant effects on working memory in healthy older adults (Linde and Alfermann, 2014; Gajewski and Falkenstein, 2018), a 6-month exercise program combining multiple fitness modalities including aerobic, strength, flexibility, balance, and coordination maintained working memory performance in older women (Klusmann et al., 2010). Meanwhile, a multi-domain exercise intervention, combining aerobic and resistance exercises, balance, and flexibility training (two 1-h on-site sessions and three 20-min home-based sessions per week), showed no significant differences in episodic memory performance in a sample of older adults who suffered from subject memory complaints or objective/clinical apparent memory impairment (Fissler et al., 2017). However, a similar 6-month multi-domain exercise intervention, which consisted of aerobic, resistance, flexibility, balance, and coordination training, enhanced episodic memory in cognitively healthy older women aged 70–93 years (i.e., Mini-Mental State Examination scores ≥26) (Klusmann et al., 2010), suggesting that variations in participant characteristics might have impacted the outcomes.

In addition to the Western-style exercise, evidence has suggested that Eastern mind-body exercise (e.g., Tai Chi Chuan/Tai Ji Quan/Taiji) has cognitive benefits in older adults (Chang et al., 2010). Most Eastern mind-body exercise includes complex coordinated sequential movements and multiple forms of exercise (e.g., aerobic fitness, muscular fitness, balance, flexibility, and coordination), as well as emotional and psychosocial components (e.g., mental concentration, breathing control, and meditation) (Chang et al., 2014). Eastern mind-body exercises have been linked to stimulating multiple aspects of cognitive functioning and have been recommended for preventing age-related cognitive decline in older adults (Chang et al., 2010). For instance, improved delayed recall on an episodic memory task has been reported in older adults with MCI after a 6-month Tai Chi intervention (Sungkarat et al., 2018). Evidence from systematic and meta-analytic research has also revealed improved performance in episodic memory in older adults with MCI (Zou et al., 2019), as well as working memory and episodic memory in older adults (Ye et al., 2021), suggesting that there are benefits of mind-body exercise on various memory domains among older adults, even among those with cognitive impairment. Neuroimaging research using resting-state functional magnetic resonance imaging (rs-fMRI) has provided further evidence showing the benefits of mind-body exercise on memory (Tao et al., 2016). For instance, intervention research using high-resolution fMRI showed that the Tai Chi group had better memory performance which was correlated with increased resting-state functional connectivity (rs-FC) between the hippocampus and medial prefrontal cortex in cognitively healthy older adults (Tao et al., 2016). While evidence has suggested benefits of exercise on memory, prior interventions have not combined both Eastern and Western-style exercise using multiple modalities. At the same time, the effects of interventions on cognitive function and brain function might vary according to the exercise modalities and the characteristics of the participants examined. Accordingly, whether an integrated intervention encompassing both Western style of physical fitness training and Eastern mind-body exercise affects memory and brain function in adults at risk for dementia remains less examined and requires further investigation.

### Other modifiable lifestyle factors, memory, and brain function

Other non-pharmaceutical interventions or modifiable lifestyle factors (e.g., meditation and social interaction) have been proposed to mitigate age-related cognitive decline (Livingston et al., 2020). Meditation (e.g., mindfulness, transcendental meditation, and Vihangam yoga), a self-regulatory technique for maintaining attention and concentration on a single aspect of sensation and focusing on the present moment (Fox et al., 2014), has been considered a cognitively stimulating activity (Gallant, 2016). Research focusing on the effects of meditation-based interventions on memory has revealed promising results (Levi and Rosenstreich, 2019). Interventional-based research comparing the effects of meditation practice on operation span test performance found improved working memory in young adults (Mrazek et al., 2013). The benefits of meditation on working memory have been reported in one recent meta-analytic study of cognitively healthy and impaired older adults aged 60 years or above (Chan et al., 2019), suggesting the potential benefits of mindfulness training for improving working memory capacity in older adults. Additionally, the beneficial effects of meditation on episodic memory have been observed (Van Vugt et al., 2012; Basso et al., 2019; Nyhus et al., 2019), suggesting the potential benefits of meditation for improving episodic memory capacity.

The long-term practice of meditation has been related to changes in memory-related brain function. Increased cortical activity in the Default Mode Network (DMN) (Froeliger et al., 2012) in experienced meditators relative to non-meditators has been reported. Findings from interventional research in older adults also suggested altered connectivity within the DMN, and between the DMN and other nodes (Cotier et al., 2017). Considering the DMN has been linked to working memory (Piccoli et al., 2015) and episodic memory (Huo et al., 2018), these findings might imply superior memory in older adults who meditate. Indeed, reviews and meta-analyses indicated that extensive meditation practice was associated with changes in the frontoparietal network and higher brain efficiency during tasks involving memory (Levi and Rosenstreich, 2019).

Social interaction might also be involved in mitigating age-related cognitive decline (Evans et al., 2019; Livingston et al., 2020). Kelly et al. (2017) conducted a meta-analysis on the associations between social relationships and cognitive functions of healthy older adults by examining the effects of different social relationships (i.e., social activities, social networks, and social support) on working memory and episodic memory, showing that greater social activity and social support were associated with superior working memory and episodic memory, respectively. Evidence for the importance of social relationships has also been supported by research examining the effects of social isolation on cognitive functioning (Evans et al., 2019), and social isolation indexed by the social network index and social activity has been correlated with poor late-life memory. Finally, findings from cohort and longitudinal research (Berkman et al., 2000; Sommerlad et al., 2019), as well as systematic reviews and meta-analyses, have suggested the detrimental effects of less social engagement on risk for dementia (Penninkilampi et al., 2018; Saito et al., 2018; Sundström et al., 2020).

Collectively, while meditation and social interaction benefit memory and enhance brain function, whether meditation and social interactions in combination with multi-domain exercise positively affect neurocognition remains unknown and requires further investigation.

### The moderators and mediators of exercise, cognitive function, and brain function

Age-related cognitive decline and late-onset AD might be influenced by several risk factors, such as the apolipoprotein E (*APOE*) gene (Martins et al., 2005). *ApoE* has three common allelic isoforms (i.e., epsilon (ε)2, ε3, and ε4). Among them, the *ApoE* ε4 allele(s) has been closely associated with cognitive impairment (Martins et al., 2005; Wang et al., 2011). The *ApoE* ε4 allele(s) is overrepresented in late-onset AD, and it has been reported that more than 48% of the AD population carries one or more *ApoE* ε4 alleles (Ward et al., 2012). *ApoE* ε4 allele(s) status has also been associated with disease progression from MCI to AD, such that the onset of AD could be reported 8–16 years earlier among individuals with the *ApoE* ε4 allele(s), compared to non-*ApoE* ε4 allele carriers (Liu et al., 2013), indicating as well even faster rates of cognitive decline (Martins et al., 2005).

The *ApoE* ε4 allele(s) might also influence the effects of exercise on cognitive functioning and the risk for AD (Hamer and Chida, 2009; Wang et al., 2011). Higher physical activity levels are more strongly associated with superior memory among older adults with one or two *ApoE* ε4 allele(s) (Smith et al., 2011). Alternatively, research has suggested a positive correlation between cardiovascular fitness levels and verbal learning memory in older adults, irrespective of the status of *ApoE* ε4 allele(s) (Boots et al., 2015). Interestingly, a positive correlation between cardiovascular fitness and performance on the Rey Auditory Verbal Learning Test was only observed in older adult men at risk for AD, indicating that there might be a gender-specific effect of cardiovascular fitness on episodic memory in older adults at risk for AD (Dougherty et al., 2017). Finally, compared to sedentary older adults with the *ApoE* ε4 allele(s), older *ApoE* ε4 carriers with higher exercise levels had better performance on the Sternberg task (Deeny et al., 2008), suggesting the benefits of exercise on working memory among those with greater genetic risk for AD. Notably, while evidence suggests that the *ApoE* genotype moderates the influence of exercise and physical fitness levels on memory, no investigation thus far has examined the role of the *ApoE* genotype in an integrated intervention composed of Western-style physical fitness training and Eastern mind-body exercise.

Exercise-evoked brain-derived neurotrophic factor (BDNF) might be a candidate biological mechanism for improving cognitive function (Miranda et al., 2019; Heinze et al., 2020). BDNF, a member of the neurotrophic family with the highest levels found in hippocampal neurons (Murer et al., 2001), has been considered a putative mediating factor with multiple aspects influencing neuronal survival and maintenance (Benarroch, 2015), as well as hippocampal-related memory formation and maintenance (Bramham and Messaoudi, 2005; Miranda et al., 2019). While BDNF is the most pervasive neurotrophin in the developed adult brain (Song et al., 2015), correlations between decreased plasma BDNF concentration, increasing age (Lommatzsch et al., 2005), and poor memory performance in healthy older adults (Erickson et al., 2010) and older adults with MCI and AD dementia (Borba et al., 2016) have been observed. Research that has explored exercise training and BDNF levels in relation to the memory has revealed improved spatial memory and increased hippocampal volume following an aerobic exercise intervention, and these exercise-induced changes in hippocampal volume were correlated with elevated serum BDNF levels (Erickson et al., 2011). Meta-analytic research has further indicated that regular exercise might not only elevate resting peripheral BDNF concentrations (Szuhany et al., 2015; Dinoff et al., 2016), but also intensify BDNF responsivity immediately after both acute and regular exercise (Szuhany et al., 2015). Notably, prior studies mainly applied a single type of exercise, leaving the effects of multi-domain exercise on BDNF and memory in older adults unknown.

### Aims of the study

**Figure 1:**
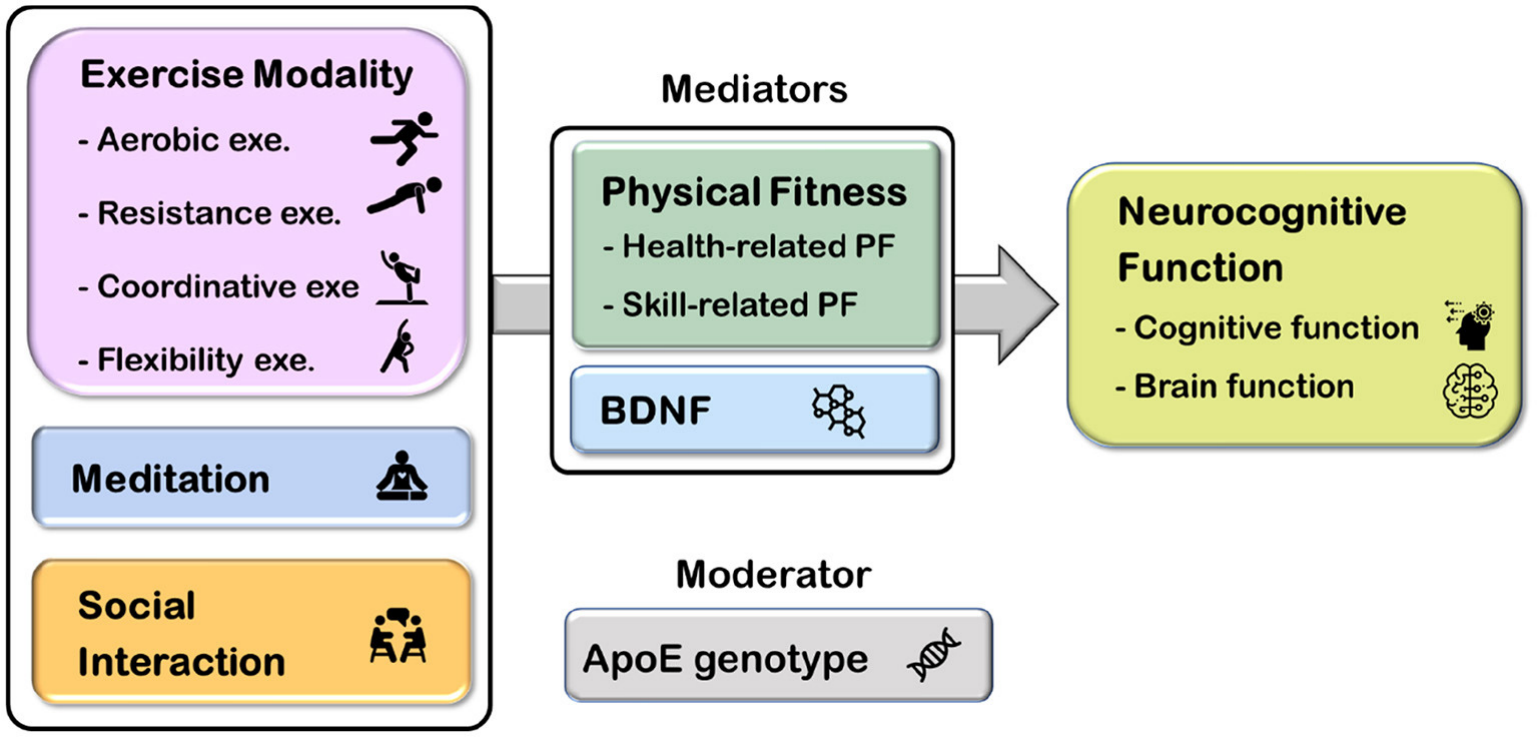
The overall picture of the Western-Eastern Brain Fitness Integration Training (WE-BFit) Trial. *ApoE*, apolipoprotein E; BDNF, brain-derived neurotrophic factor; PF, physical fitness; exe., exercise.

Several studies have examined the independent effects of chronic exercise, meditation, or social interaction on memory and brain health; however, research has yet to examine the influence of multi-domain exercise with the consideration of moderators and mediators on memory in adults at risk for AD. The current study entitled Western-Eastern Brain Fitness Integration Training (WE-BFit) Trial aims to utilize a randomized controlled design to examine the effects of a 6-month multi-domain exercise program combining multiple exercise modalities (i.e., aerobic exercise, resistance exercise, coordinative, and flexibility exercise), meditation, and social interaction on working memory and episodic memory, as well as brain function, in cognitively healthy late middle-aged and older adults. Additionally, whether the effects of this intervention will be affected by *ApoE* genotype, the status of physical fitness, or neurotrophic changes will be further examined (Figure 1).

## Methods

### Study design and schedule

**Figure 2:**
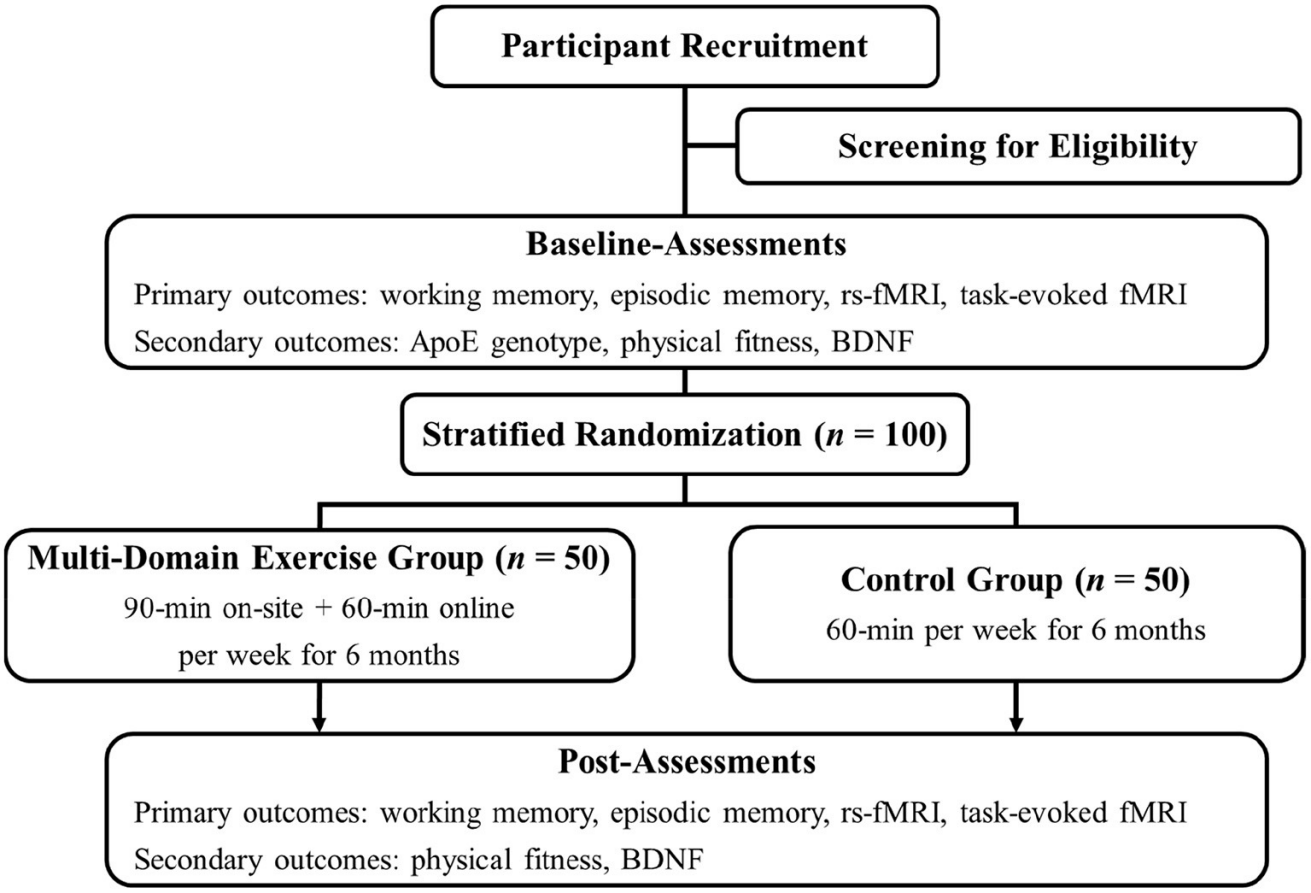
Study flowchart. *ApoE*, apolipoprotein E; BDNF, brain-derived neurotrophic factor; fMRI, functional magnetic resonance imaging; rs-fMRI, resting-state functional magnetic resonance imaging.

The multi-domain interventional trial of WE-BFit is designed as a double-arm, randomized controlled trial (RCT) targeting potential factors for promoting working memory, episodic memory, and brain function in cognitively healthy late middle-aged and older adults who are and who are not at genetic risk for AD. The trial is planned to start in April 2022 and aims to recruit 100 eligible participants who will be randomly assigned into two parallel groups (i.e., the multi-domain exercise group, *n* = 50; the control group, *n* = 50). *ApoE* genotypes of eligible participants will be examined at the baseline assessment. Additionally, other primary outcomes (i.e., working memory, episodic memory, and brain function) and secondary outcomes (i.e., multiple components of physical fitness and BDNF) will be assessed before and after the 6-month intervention. This trial will be led primarily by the Faculty of Physical Education and Sport Sciences of the National Taiwan Normal University. A flow chart of the current trial is presented in Figure 2.

### Participants

To ensure a sufficient sample size, the sample size calculation for the current study was conducted using G^*^Power version 3.1.9.4 (ANOVA; repeated measures, within-between interaction). Multiple recruitment strategies will be utilized, including online social media advertising and word-of-mouth referrals, as well as posters and flyers placed or distributed in local community centers and organizations. Finally, individuals from previous studies will also be screened for recruiting potential participants. Before participating in the current study, potential participants will be screened by phone, followed by an on-site interview to ensure they meet the inclusion criteria.

Eligible participants (half of them are *ApoE* ε3/ε4 or *ApoE* ε4/ε4 carriers) will be provided sufficient information, and informed consent relating to the study will be provided prior to the initiation of the study. Recruitment, enrollment, and randomization will occur on a rolling basis. Finally, a total of 100 community-dwelling, cognitively healthy adults aged 45–70 years who are able to participate in moderate-intensity exercise will be recruited in a 1-year time period in Taipei, Taiwan. The inclusion and exclusion criteria are detailed in Table 1.

**Table 1:** Inclusion and exclusion criteria for participating in the study.

**Inclusion criteria**	**Exclusion criteria**
- Adults aged from 45 to 70 years	- Diagnosed or self-reported cognitive problems (e.g., mild cognitive impairment or dementia)
- Normal or corrected-to-normal vision	- The diagnosed or self-reported physical disease (e.g., untreated hypertension and chronic heart disease, stroke, brain tumor, musculoskeletal disorders, other exercise contradictions)
- Able to speak and read Chinese	- Diagnosed or self-reported major psychiatric illness (e.g., major depression, schizophrenia)
- Scores of MMSE ≥ 25	- Diagnosed or self-reported neurodegenerative disease (e.g., AD and other dementias, PD and PD-related disorders, Huntington's disease)
- PAR-Q score = 0	- History of severe alcohol or drug abuse
- Able to conduct the exercise with moderate intensity	- History of chemotherapy
- Meet the criteria to undergo MRI	- Unable to complete the MRI scan
	- Traveling consecutively for 3 weeks or more during the study
	- Unwillingness to be randomized to one of the two groups
	- Currently participating in another study trial

AD, Alzheimer's disease; MMSE, Mini-Mental Status Examination; MRI, magnetic resonance imaging; PAR-Q, Physical Activity Readiness Questionnaire; PD, Parkinson's disease.

### Randomization and blinding

To minimize the potential bias of individual differences or covariates, eligible participants will be recruited and randomly assigned to either a multi-domain exercise group or an online educational courses control group with a 1:1 allocation ratio using a computerized permuted block randomization algorithm with stratification (Lim and In, 2019). Additionally, the randomization will be stratified by *ApoE* genotype (High-risk: *ApoE* ε3/ε4 and *ApoE* ε4/ε4 vs. Low-risk: *ApoE* ε2/ε2, *ApoE* ε2/ε3, *ApoE* ε2/ε4, and *ApoE* ε3/ε3) to confirm equal allocation to both groups based on these criteria.

The randomization list for the assignment sequence will be created using the IBM SPSS Statistics for Windows (SPSS Inc., Chicago, IL, USA). Except for staff involved in the multi-domain exercise program and online educational program, assessors and data analyzers will be kept blind to participants' group assignments.

### Intervention protocols

Eligible individuals will participate all together in either the multi-domain exercise group or the control group for 6 months.

#### Multi-domain exercise group

The exercise program for the multi-domain exercise group is designed by senior academic psychologists with input from a team of professional exercise experts experienced in working with late middle-aged and older adults. The multi-domain exercise group consists of a 6-month intervention, including one 90-min on-site and several online sessions, for up to 60 min per week. The program primarily focuses on multiple exercise modalities (i.e., aerobic exercise, resistance exercise, and coordinative exercise), with two additional lifestyle domains (i.e., meditation and social interaction). Specifically, each exercise course includes five stages:

1. Stage 1: 15 min of warm-up and aerobic exercise.
2. Stage 2: 25 min of resistance and flexibility exercise from the “Western exercise” perspective.
3. Stage 3: 30 min of the main exercise, named as Bagua Daoyin, consisting of resistance exercise, coordination, and flexibility exercise from the “Eastern exercise” perspective. This exercise, including eight forms of sequential movements, emphasizes muscular strength and endurance of the upper and lower trunk, trunk rotation, weight shifting, and coordination of visual and musculoskeletal systems. Additionally, awareness of the proprioception with mental focus from different parts of the body will be heightened.
4. Stage 4: 10 min of social interaction exercise. The exercise program will take place in a group format, and practice in pairs or small groups will be frequently encouraged, providing sufficient social interactions among participants.
5. Stage 5: 10 min of cool down and meditation. After the cessation of the main exercise session, a cool down and mindfulness-based meditation will be conducted. The mindfulness-based meditation is based on a mindfulness program utilized previously, focusing on the components of mindful breathing, awareness, nonjudgement, and acceptance (Nien et al., 2020).

Each on-site exercise course will be conducted at one recreational center in Taipei, Taiwan, supervised by experienced exercise instructors, who will instruct the movements designed for the multi-domain exercise program.

#### Control group

The control group will not receive any exercise program and will be informed to maintain their lifestyles. In addition, participants will be invited to attend one 60-min online group educational course regarding the effects of exercise on cognitive function and general instructions for cognitive promotions per week to eliminate the experimental expectation effect. The participants will be required to provide their physical activity behavior once per month for 6 months (see Figure 2). After data collection at the Post-Assessments, the participants can voluntarily join the multiple-domain exercise program for the next 3 months as compensation for their participation.

### Assessments and outcome measures timeline

**Figure 3:**
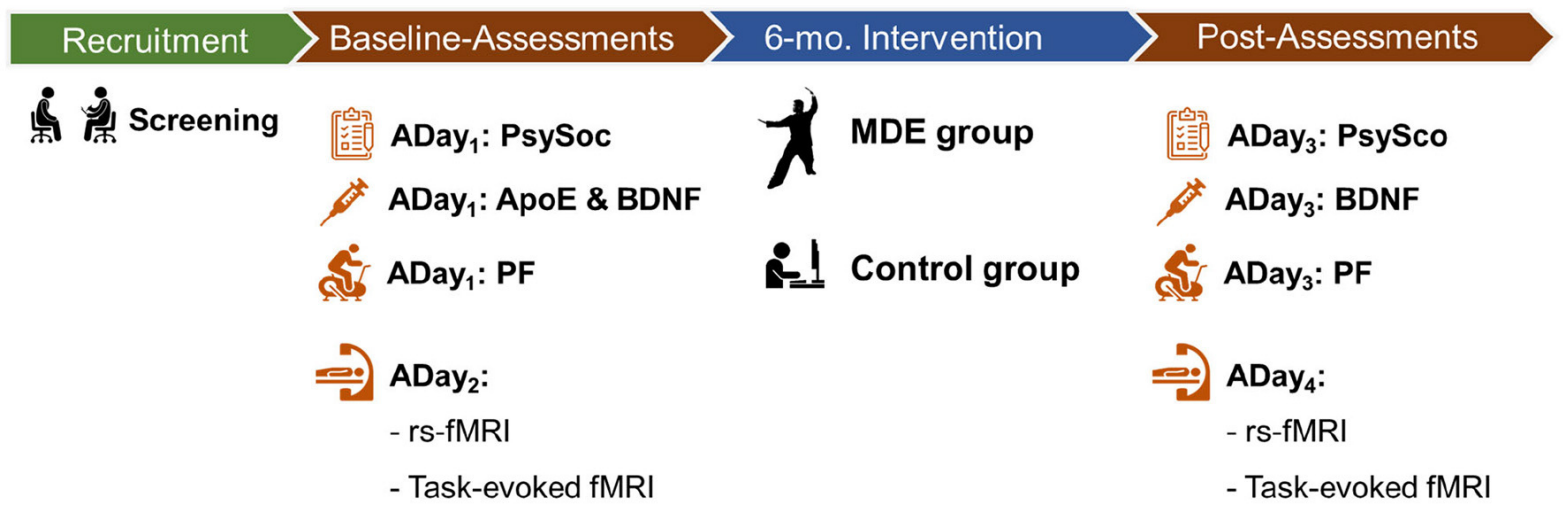
Experimental timeline. ADay_1_, ADay_2_, ADay_3_, and ADay_4_, assessment day 1 to assessment day 4, respectively; BDNF, brain-derived neurotrophic factor; fMRI, functional magnetic resonance imaging; MDE, multi-domain exercise; PF, physical fitness assessments; PsySoc, psychosocial measures; rs-fMRI, resting-state functional magnetic resonance imaging.

All participants will undergo multiple assessments before (Baseline-Assessments) and after (Post-Assessments) the intervention. The Baseline-Assessments will be organized into two assessment days (ADay) in the following order: (1) ADay_1_: psychosocial measures, blood extraction, and physical fitness assessment, and (2) ADay_2_: fMRI scans. The same assessment order (ADay_3_ and ADay_4_) will be carried out in the Post-Assessments. Assessments will be carried out at National Taiwan Normal University, and fMRI scans will be performed in the Imaging Center for Integrated Body, Mind, and Culture Research in National Chengchi University, Taipei, Taiwan, within 3 weeks of completion of the initial screening and the cessation of the 6-month intervention (Table 2). The timeline for the trial is visualized in Figure 3.

**Table 2:** Assessments.

	**Recruitment**	**Baseline-assessments** **(**<**3 weeks before the intervention)**		**6-month intervention**			**Post-assessments** **(**<**3 weeks after the** **intervention)**	
	Screening	**ADay**_**1**_	**ADay**_**2**_	Initial intervention	Middle intervention	Final intervention	**ADay**_**3**_	**ADay**_**4**_
Information of study	**X**							
Review of criteria	**X**							
Medical history, General health status, MMSE, PAR-Q	**X**							
Informed consent form	**X**							
Battery of psychosocial measures	**X**						**X**	
Blood extraction		**X**					**X**	
Battery of physical fitness tests		**X**					**X**	
Magnetic resonance imaging			**X**					**X**
rs-fMRI								
fMRI: n-back task, RISE task								
Attendance				**X**	**X**	**X**		

ADay, Assessment days; MMSE, Mini-Mental Status Examination; PAR-Q, Physical Activity Readiness Questionnaire; rs-fMRI, resting-state functional magnetic resonance imaging; RISE, relational and item-specific encoding.

### Primary outcome assessments

#### N-back working memory task

A modified n-back working memory task (Li et al., 2014) will be programmed using E-Prime to examine participants' working memory. The n-back task contains a sequence of single-digit numbers (i.e., 1–9) presented with a duration of 500 ms and a fixed inter-stimulus interval of 2,000 ms. All participants will be instructed to respond if the current stimulus matches the one from *n* steps earlier in the sequence. In addition, all participants will be required to respond by pressing a button on a standard keyboard when the stimulus matches and pressing another button when the stimulus does not match. Both 1-back and 2-back conditions will be included in the task. A total of four blocks with 16 trials will be conducted, in which the stimuli will appear in random order. The total task time is about 7 min, and reaction time and accuracy will be recorded as indices of behavioral performance.

#### Relational and item-specific encoding task

The computerized Relational and Item-Specific Encoding (RISE) task is modified from the original RISE task (Ragland et al., 2012, 2015; Erickson et al., 2019) and will be utilized to assess episodic memory. Briefly, the task consists of two phases. During the first phase (i.e., the encoding phase), pairs of item-specific objects or relational objects will be alternatively presented. Participants will be instructed to identify whether the object is living or nonliving (item-specific encoding response trials) or whether one item could fit inside the other item in real life (relational encoding response trials). During the second phase (i.e., the recognition phase), participants will determine whether the item has been presented previously or never presented (item recognition). Stimuli will be presented for 3 and 2 s for the encoding phase and item recognition phase of the task, respectively, and participants are encouraged to respond as quickly and accurately as possible. The total task duration will be ~30 min. Reaction time and accuracy during both encoding and recognition phases will be recorded as the indices of behavioral performance.

#### Functional magnetic resonance imaging acquisition and analysis

All participants will undergo a series of whole-brain functional magnetic resonance imaging (fMRI) before and after the intervention. The data acquired will include resting-state fMRI (rs-fMRI) and task-evoked fMRI using a Siemens 3.0 T MRI scanner (Magnetron Prisma, Siemens, Germany) with a 32-channel head coil at the Imaging Center for Integrated Body, Mind, and Culture Research in National Chengchi University, Taipei, Taiwan. The parameters for MRI screening will be adapted from previous studies (Castells-Sánchez et al., 2019; Erickson et al., 2019).

##### The resting-state fMRI scan session (rs-fMRI)

A single-shot T2^*^-weighted gradient echo-planar image (EPI) sequence will be applied (TE/TR/flip angle = 30 ms/2,000 ms/90°, 64 contiguous axial slices with a slice thickness of 3.0 mm).

##### Task-evoked fMRI scan sessions (task-evoked fMRI)

Participants will be instructed to perform two fMRI tasks during the task-evoked fMRI scans. The two fMRI tasks will include n-back working memory task and the RISE task as described above. A single shot T2^*^-weighted gradient EPI sequence will be applied to the two fMRI tasks with the following parameters: TE/TR/flip angle = 30 ms/2,000 ms/90°, and 64 contiguous axial slices will be acquired with a slice thickness of 3.0 mm.

### Secondary outcome assessments

#### Cardiovascular fitness

All participants will be informed to refrain from high-intensity exercise for 8 h and from eating or drinking any beverage for 2 h before the cardiovascular fitness test. Participants will be asked to wear the heart rate (HR) monitor (Polar HR monitor, Mode V800, Finland) to monitor their HR during the entire testing protocol. Cardiovascular fitness will be assessed by the YMCA cycling ergometer test (Golding et al., 1989), which is a widely recommended way to estimate maximal oxygen uptake (i.e., VO_2max_) for adults with Class A risk stratifications (Fletcher et al., 2001). The YMCA cycling ergometer test comprises two to four 3-min consecutive stages on an electronically braked cycle ergometer (Corival IV CPET, Lode, Netherlands) and is targeted to reach the participants' HR according to their 85% age-predicted maximal HRs [HR_max_ = 206 – (0.67 × age_years_) (Gellish et al., 2007)]. Specifically, participants are instructed to pedal at a speed of 50 rpm throughout the test. In the initial stage, participants are asked to cycle at a workload of 150 kgm/min (25 W) for 3 min. The workloads of the following two stages will depend on each participant's HR recorded during the second and third minute of the initial stage (e.g., HR <80 bpm, the workloads for the second and third stages will be 600 kgm and 750 kgm, respectively; HR >100 bpm, the workloads for second and third stages will be 300 kgm and 450 kgm, respectively). The process will be terminated if the participant's HR reaches their 85% age-predicted maximum HR. Finally, VO_2max_ of each participant will be estimated based on the slope of HRs, the workload, and their body weight.

#### Other fitness indices

In addition to cardiovascular fitness, other indices associated with health-related physical fitness (i.e., body composition, muscular fitness, and flexibility) and skill-related physical fitness (i.e., balance and power) will be assessed.

Regarding health-related physical fitness, body composition [e.g., body water, body fat, skeletal muscle mass, body mass index (BMI), and percentage of body fat] will be assessed using a multifrequency, whole body, and segmental body composition analyzer (ACCUNIQ BC380 Body Composition Analysis, SELVAS Healthcare Inc., Daejeon, Korea) with bioelectrical impedance analysis (BIA) technology. Compared to the reference values of dual-energy x-ray absorptiometry, the measuring device demonstrates high correlation coefficients for lean body mass (kg) in Asian men and women (*r* = 0.983 and *r* = 0.957, respectively), as well as high correlation coefficients for percent of body fat in Asian men and women (*r* = 0.881 and 0.893, respectively) (Yang et al., 2018). Each measurement will take around 2 min. Muscular fitness will be assessed using push-ups, and the scores will be recorded as the number of push-ups completed in 30 s. Finally, flexibility will be assessed using the sit-and-reach test, in which participants will be instructed to reach as far as possible with their palms facing down while sitting down on the floor with legs stretched straight out. Further distance indicates better lower back and hip joint flexibility.

As for skill-related physical fitness, participants' balance will be assessed using the Single-Leg Stand (30 s) assessment. Participants will be instructed to perform the test three times with and without their eyes closed. The length of time the participant can maintain their balance will be recorded as the index of balance performance. Finally, the participant's leg power will be assessed using the distance of the Standing Long Jump. The length between the takeoff line and the nearest point of contact on the landing will be recorded as the power performance index.

#### Genotype and blood assays

##### *ApoE* genotype

Blood samples from the antecubital veins will be collected by licensed medical technicians/nurses during participants' first visit to the laboratory before the random assignment. The genotypes will be determined using a polymerase chain reaction method with modification. Two (rs429358: *ApoE* C112R; rs7412: *ApoE* R158C) genes will determine identification of the *ApoE* allele(s) (*ApoE* ε2, *ApoE* ε3, and *ApoE* ε4). Based on the genotypes, participants will be categorized as high risk (*ApoE* ε3/ε4 and *ApoE* ε4/ε4) or low risk (*ApoE* ε2/ε2, *ApoE* ε2/ε3, *ApoE* ε2/ε4, and *ApoE* ε3/ε3) for AD occurrence.

##### Neurotrophic measure BDNF

All participants will be asked to avoid exercising 8 h before the blood test. Following an overnight fast, participants' blood antecubital veins will be drawn by licensed medical technicians/nurses during the Baseline- and Post-Assessments. Approximately, 6 ml of venous blood will be collected by vacutainers (CAT, BD Vacutainer). The blood will then be separated using a centrifuge at 3,000 rpm for 15 min, and the supernatant fluid will be stored at −80°C for serum marker assays. The peripheral serum BDNF levels will be assayed using a ChemiKineTM BDNF Sandwich ELISA Kit (Millipore, Billerica, MA, USA). The procedure has been employed in our previous studies (Chang et al., 2017a).

#### Psychosocial measures

##### Mindfulness

Mindfulness will be assessed by the Chinese version of the Mindful Attention Awareness Scale (CMAAS) (Nien et al., 2020). The CMAAS (Chang et al., 2011) was based on the Mindful Attention Awareness Scale (MAAS) (Brown and Ryan, 2003). The MAAS assesses levels of dispositional mindfulness. The CMAAS is a 15-item questionnaire. Participants respond on a 6-point Likert scale from 1 (almost always) to 6 (almost never). Higher scores are associated with higher dispositional mindfulness. The CMAAS has been shown to have high internal consistency, with Cronbach's alphas at 0.88.

##### Other psychosocial measures

Potential confounding factors associated with psychosocial factors will be assessed using the following questionnaires: sleep *via* a Chinese version of the Pittsburgh Sleep Quality Index (Tsai et al., 2005) and the Chinese version of short forms of the Geriatric Depression Scale (GDS-15) (Sheikh and Yesavage, 1986; Liu et al., 1998), and health-related quality of life *via* the WHOQOL-OLD-Taiwan (Yao et al., 2017).

### Retention and adherence

To maximize participants' adherence throughout the entire study period, participants will be frequently contacted by the investigators or research staff *via* telephone and e-mail. Participants will also be encouraged to contact the research staff or leave messages if they have any inquiries or concerns about the study. Participants will receive financial reimbursement for each of the assessments (~20 US dollars per hour).

### Power analysis

The study will recruit a total sample size of 100 participants for final statistical analyses. The sample size is estimated from an *a priori* power analysis using G^*^Power 3.1.9.4 (power = 0.80, alpha = 0.05) and the effect size (Hedges' *g* = 0.30) based upon our previous meta-analysis that examines the effects of exercise interventions on executive function, including working memory in adults of age 55–65 years (Chen et al., 2020a).

### Statistical analysis

To address whether the 6-month multi-domain exercise program affects working memory or episodic memory performance and brain functioning, we will employ a two-way repeated-measure analysis of variances (ANOVA) of mixed design with group status (i.e., multi-domain exercise group vs. control group) as the between-subject variable and time points (i.e., Baseline-Assessment vs. Post-Assessment) as the within-subject variable, with the Greenhouse–Geisser correction, where deemed appropriate. The two-way ANOVA will be employed individually for each primary outcome. Multiple *t*-test comparisons will be conducted as follow-up by setting the familywise alpha levels at 0.05, prior to a Bonferroni correction.

To address the moderating role of *ApoE* genotype on the effect of the multi-domain exercise program on working memory and episodic memory performance, as well as brain function assessed from neuroimaging metrics, we will employ the three-way repeated-measure ANOVA of mixed design with group status as the between-subject variable, and *ApoE* genotype [i.e., high-risk candidates (*ApoE* ε3/ε4 and *ApoE* ε4/ε4) vs. the low-risk candidates (*ApoE* ε2/ε4, *ApoE* ε2/ε3, *ApoE* ε2/ε2, and *ApoE* ε3/ε3)] and time points as the within-subject variable, with a Greenhouse–Geisser correction where deemed appropriate. In addition, multiple *t*-test comparisons will be conducted as follow-ups by setting the familywise alpha levels at 0.05 prior to a Bonferroni correction.

To address whether the effects of the multi-domain exercise program on working memory, episodic memory, and brain function are mediated by physical fitness and BDNF, we will employ Pearson product-moment correlations to examine relationships between change in physical fitness, BDNF, and the primary and secondary outcomes. Separate mediation analyses will be conducted using PROCESS software for SPSS. Statistical significance of mediators will be considered if the 95% bias-corrected bootstrap confidence interval (5,000 bootstrap samples) does not include zero. For all analyses, age, sex, BMI, and educational levels will be controlled for, and the alpha value will be set at 0.05, prior to statistical adjustment for all analyses.

### Ethics statement

The protocol has been proven by the Institutional Review Board of the National Taiwan Normal University, Taiwan (REC number: 20212HM023) and has been registered on ClinicalTrials.gov (NCT05068271). All participants will be given informed consent according to the Declaration of Helsinki; the purpose, methodological approaches, and potential risks of the current study will be fully explained prior to participating in the study.

## Discussion

Aging has been associated with cognitive decline and an increased risk for AD, as well as deterioration of brain function (Salthouse, 2019; Li et al., 2020). Since the curative effects of pharmaceutical interventions are limited (Mehta et al., 2017), identifying cost-effective non-pharmaceutical intervention strategies for preserving memory and brain function has been prioritized in the field. Notably, while several factors (e.g., exercise, physical fitness, meditation, and social interaction) have been described as beneficial for cognitive and brain function, no multi-domain intervention trials combining the Western style of physical fitness training and the Eastern mind-body exercise components of meditation and social interaction have been conducted for the prevention of age-related decline in memory and brain function in late middle-aged and older adults with and without a genetic risk for AD.

Accordingly, this WE-BFit trial will be the first RCT to evaluate the effectiveness of this type of intervention for promoting memory and brain function in this population while considering the moderators and mediators (e.g., *ApoE* genotype, BDNF, and physical fitness). The results of WE-BFit could provide valuable insight regarding the effectiveness of different components of an intervention program on cognitively healthy late middle-aged and older adults, such as the number of sessions per week, the length of each session, and the aspects of the exercise program (e.g., exercise modalities, meditation, and social interaction).

Several challenges of this study are worth mentioning. For instance, the current trial will recruit 50 cognitively healthy participants with a genetic risk for AD. Nevertheless, the recruitment of sufficient participants is challenging, given that the prevalence of *ApoE* ε4 allele(s) in Taiwan is only around 20% (Hong et al., 1996; Wang et al., 2011). Unfortunately, this challenge will be further escalated by the occurrence of the COVID-19 pandemic. Although this challenge might be partially alleviated through recruiting *ApoE* ε4 allele(s) carriers from our previous research and the progressive ease of the COVID-19 pandemic in Taiwan, we still expect the inclusion of a sufficient number of participants, especially the *ApoE* ε4 allele(s) carriers, will be difficult. Drop-out and adherence will be other challenges in the current study. Two strategies will be applied to curtail these challenges. First, frequent contacts and regular newsletters will be delivered to all participants from the research group. Furthermore, the multi-domain exercise group can attend two out of 10 optional exercise sessions each week instead of adhering to a fixed exercise schedule. These approaches enhance the personal connection to the study and provide user-friendly access to the exercise program, and we expect this to enhance participants' adherence and minimize the risk of them withdrawing from the intervention (Robiner, 2005). Finally, the current trial collects a considerable amount of outcome measures, and many of them have to be collected in a time-sensitive manner. For instance, to accurately reflect the relationships between physiological conditions and brain health, physical fitness, BDNF levels, cognitive tasks, and fMRI assessments will be completed within 3 weeks before and after the 6-month exercise program, respectively. To overcome this potential challenge, a limited number of participants (e.g., five people) will be grouped as one intervention unit, and the unit will coordinate the most suitable time for initiating the assessments and exercise program.

In summary, the WE-BFit program will offer significant insight into the health and societal impacts of a multi-domain exercise program (e.g., Western style of physical fitness training and Eastern mind-body exercise, meditation, and social interaction) on memory and brain function in late middle-aged and older adults. This study will provide valuable results informing healthcare professionals, gerontology investigators, and healthcare policymakers about the effectiveness of a non-pharmaceutical intervention on cognitive and brain function.

## Ethics statement

The studies involving human participants were reviewed and approved by Institutional Review Boards from the National Taiwan Normal University. The patients/participants will provide their written informed consent to participate in this study.

## Author contributions

Conceptualization: Y-KC, KE, S-HC, C-MH, and C-HC. Methodology: KE, F-TC, R-HL, J-RS, and C-HC. Writing: Y-KC, SA, F-TC, S-HC, C-MH, and C-HC. Visualization: R-HL, J-RS, and S-HC. Supervision: Y-KC, KE, and C-MH. Reviewing and editing: Y-KC, KE, SA, F-TC, R-HL, J-RS, S-HC, C-MH, and C-HC. All authors contributed to the article and approved the submitted version.

## Funding

This work was supported by part of a grant from the National Science and Technology Council (110-2410-H-003 -142 -MY3; MOST 111-2628-H-003-009) and National Taiwan Normal University from the Higher Education Sprout Project by the Ministry of Education (MOE) in Taiwan to Y-KC.

## Conflict of interest

The authors declare that the research was conducted in the absence of any commercial or financial relationships that could be construed as a potential conflict of interest.

## Publisher's note

All claims expressed in this article are solely those of the authors and do not necessarily represent those of their affiliated organizations, or those of the publisher, the editors and the reviewers. Any product that may be evaluated in this article, or claim that may be made by its manufacturer, is not guaranteed or endorsed by the publisher.
